# A Modified Impedance-Frequency Converter for Inexpensive Inductive and Resistive Sensor Applications

**DOI:** 10.3390/s19010121

**Published:** 2019-01-02

**Authors:** Michał Nowicki

**Affiliations:** Warsaw University of Technology, Institute of Metrology and Biomedical Engineering, 02-525 Warsaw, Poland; nowicki@mchtr.pw.edu.pl; Tel.: +48-690-650-386

**Keywords:** inductive sensor, resistive sensor, transducer, Arduino

## Abstract

In this paper an exceptionally simple transducer is presented that is developed for experimental and custom-made sensors with inductive or resistive impedance output. It is based on a venerable 555 Integrated Circuit in a modified astable configuration. Due to single supply 5 V operation, it is directly compatible with most modern microcontroller systems, such as the popular Arduino platform. Various exemplary sensor characteristics are presented, including displacement, force, magnetic field, temperature and light sensing applications. While the transducer is not designed for high accuracy, it allows for fast and inexpensive application of various experimental sensors, such as magnetoelastic or GMI (Giant Magneto Impedance) sensors.

## 1. Introduction

**Figure 1:**
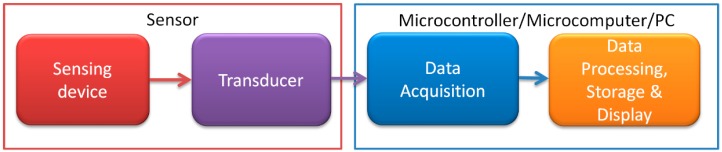
Simplified measurement chain schematic.

In recent years, there has been an unprecedented R&D increase in both new sensor designs [1,2,3,4,5] and simplified microcontroller systems, such as Arduino [6]. However, there is a gap between new, experimental sensor output signals and standardized microcontroller inputs. It is most evident for inductive sensors, where much additional design work would be needed to procure outputs such as an 0–5 V DC output signal. Examples of such inductive sensors include magnetoelastic tensile, compressive and torsional [7,8,9,10,11,12,13] devices, as well as various GMI (Giant Magneto Impedance) [14,15,16,17] and Stressimpedance sensors [18,19,20]. Thus, there is still a place for the development of intermediate systems, i.e., sensor transducers (Figure 1).

It is actually easiest to convert inductance changes into a frequency signal, which is described commonly as a hybrid analog/digital type signal, as it is directly measurable with digital inputs of most microprocessors. Frequency signals also have additional benefit, such as high resistance to external interference/distortion, and long cable runs being possible.

Typical inductance-frequency converters, used for example with magnetoelastic sensors [21], are LC oscillators utilizing FET/BJT transistors or Operational Amplifiers, such as the Colpitts type oscillator [22]. There are also some high precision impedance-frequency transducers using quartz crystals which compensate for temperature drift, and have fast response [23,24]. The greatest advantage of LC oscillators is that the response can be calculated simply as:

(1) $$f=\frac{1}{2\pi \sqrt{\mathrm{LC}}}$$

They have some significant drawbacks however—the quality factor *Q* of LC tank circuit must be high enough for the circuit to oscillate. Therefore, the losses in the inductive sensor must be low. Additionally, the Op-Amp version needs symmetrical power supply, which raises costs considerably, and prohibits direct plug-and-play in Arduino systems.

In order to overcome these problems, a new transducer is presented, based on venerable 555 IC in astable configuration. The circuit was significantly modified in order to output near 50% duty cycle square wave, with frequency determining elements limited to one capacitor C and one impedance Z, in contrast with commonly used astable configurations needing one capacitor and two similar resistors. Its greatest advantage in comparison with other solutions is the possibility of utilization of sensors with various kinds of impedance, from purely resistive to near-ideal inductive ones. 

## 2. Materials and Methods

### 2.1. New Transducer

**Figure 2:**
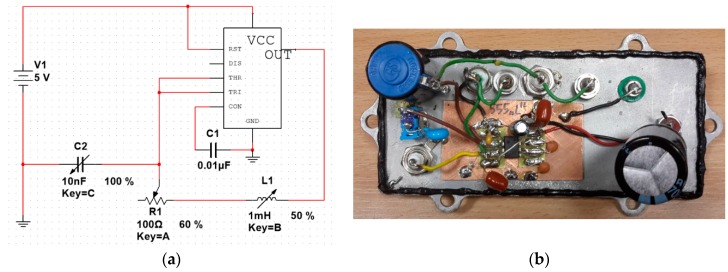
(**a**) Astable 555 circuit sensitive for impedance changes. Element L1 is the sensor’s impedance, connected in the feedback loop to the frequency range setting capacitor C2. This capacitor is chosen experimentally to obtain ~100 kHz transducer output for the given sensor. R1 is series variable resistance, which can be adjusted to tune the transducer characteristic—it is not needed for most applications. (**b**) Photo of prototype unit with additional input/output connectors and selectable range setting capacitors C2.

The impedance-frequency converter is based on modified 555 integrated circuit in an astable configuration. Figure 2 presents a schematic diagram of the proposed circuit.

The schematic is a heavily modified version of standard 555 astable oscillator circuit with frequency-setting resistor substituted for variable impedance (sensor output). It eliminates most of the output signal stability problems, and allows for fine-tuning of the output characteristics. The output signal is square-wave with approximately 50% duty cycle. Furthermore, in standard configuration two resistive elements (preferably identical) are needed, while the presented circuit needs one, which simplifies its use.

The transducer accepts a wide range of input impedances, from purely resistive to inductive. It opens the possibility of straightforward application of various sensors for e.g., Arduino-based systems. Most of the sensor characteristics are nonlinear, thus microcontroller software is needed to calculate measured quantity. While the transducer operates on a wide range of supply voltages, all of the presented sensors were driven by 5 V supply, to further justify its direct compatibility with modern microcontroller systems. 

The experimental transducer was constructed according to the schematic in Figure 2, within a shielded enclosure, and with provisions for C2 and R1 adjustment. The L1 (the impedance-changing sensor) was connected with standard BNC connectors and coaxial cables to minimize electrical interference.

The cost of presented transducer as compared with other possible solutions is favorable, especially for inductive sensors. The 555 IC plus components and board is about 1 € +3 € for PCB manufacture and shipping costs. The cost of a 16 bit ADC module is about 5–10 €, and is suitable only for resistive sensors in voltage divider configuration. The inductive sensors need LC circuits, and the main cost is the additional symmetrical power supply for operational amplifiers—good quality ones will cost about 30 €, and there is still the cost of PCB and other components involved. 

### 2.2. Exemplary Sensors

**Figure 3:**
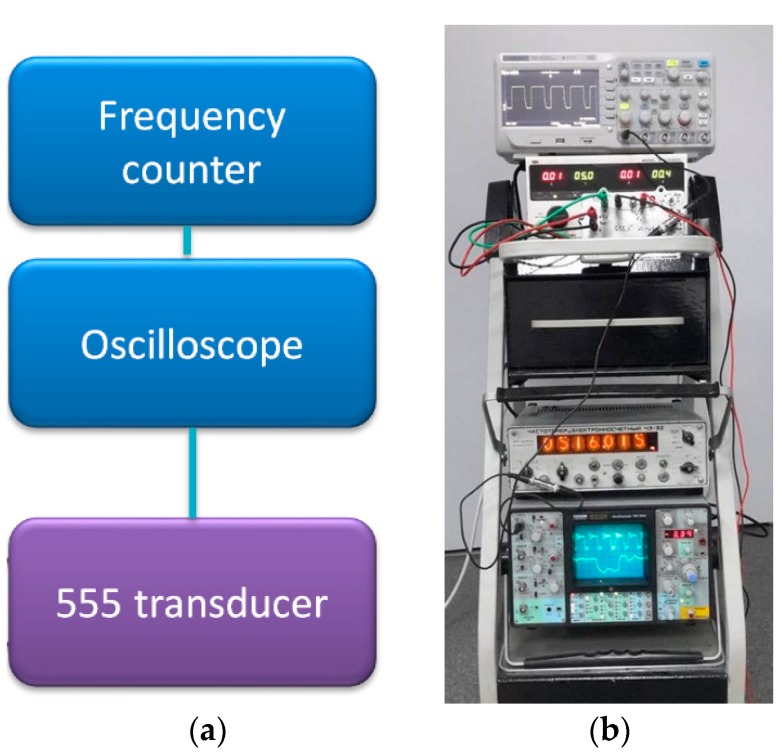
(**a**) Schematic diagram of mobile measurement test stand for a 555-based transducer, utilized for various sensors calibration; (**b**) photograph of actual test stand with power supply and additional digital oscilloscope.

The transducer operation was verified on various experimental sensors, from inductive to resistive ones. In all of the presented test stands, the 555 transducer was connected to a Schlumberger oscilloscope (model 5228, Schlumberger/Sefram, St Etienne, France) for waveform monitoring and a Ч3-32 frequency counter (Moscow, Russia) for frequency measurements (Figure 3). The frequency measurement uncertainty was negligible in comparison with transducer thermal stability and measured values uncertainties. All of the presented sensors had constructional materials costs that were under 1 €. 

#### 2.2.1. Inductive Displacement Sensor

**Figure 4:**
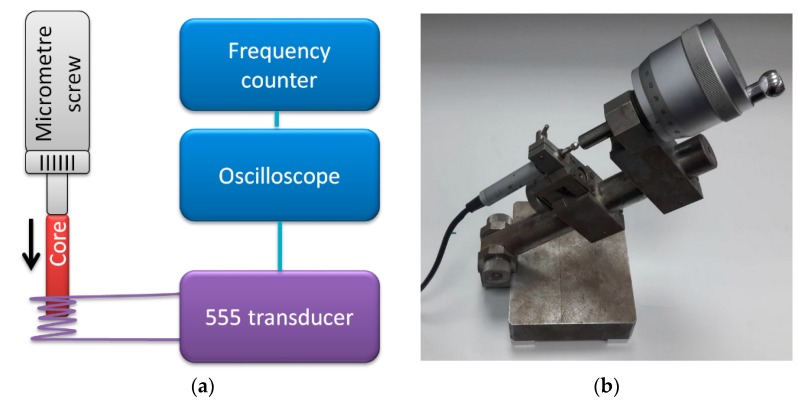
(**a**) Schematic diagram of measurement test stand for inductive displacement sensor calibration; (**b**) photograph of inductive sensor in mount with micrometer screw.

The transducer operation was verified on moving-core inductive displacement micrometer sensor. The schematic diagram of the measurements test stand is presented in Figure 4.

The sensor consisted of a coil and moving iron core, which allowed for an impedance change with the core’s displacement. The coil was connected as impedance in the 555 transducer circuit. Displacement was set with micrometer screw with 0.5 μm uncertainty. 

#### 2.2.2. Magnetoelastic Force Sensor

**Figure 5:**
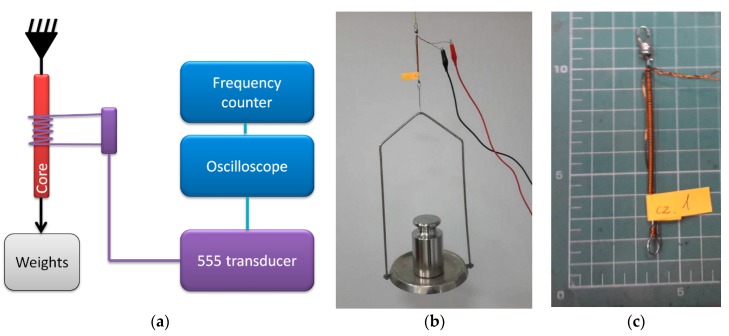
(**a**) Schematic diagram of measurement test stand for magnetoelastic force sensor calibration; (**b**) photo of measurement stand; (**c**) photo of developed magnetoelastic sensor.

The transducer operation was verified further on a simplistic magnetoelastic force sensor. The schematic diagram of the measurements test stand is presented in Figure 5. The original 555 transducer idea comes from the ongoing investigation on magnetoelastic sensor principles, and question whether high-frequency impedance change can be substituted for resistance change in 555 astable circuits.

In this configuration, the 555 transducer was connected to the coil wound on an ST3 constructional mild steel wire, 1.5 mm diameter, 100 mm long. The coil was wound uniformly on middle 80 mm, and consisted of 200 windings. The wire was loaded with calibrated laboratory weights. Thus, both the force set and the frequency measurement errors were negligible in comparison with transducer thermal stability. 

Due to the tensile stresses, the permeability of the steel core changes [25,26], which changes the coil impedance and in turn affects the 555 transducer output frequency. A similar sensor could be used in bending or torsional [27] mode of operation, with significant sensitivity, but different characteristics. Amorphous [28] or ferrite [11] based magnetoelastic sensors are also suitable, the limiting condition being the needed driving current, which has to be supplied through the 555 IC.

#### 2.2.3. GMI Magnetic Field Sensor

**Figure 6:**
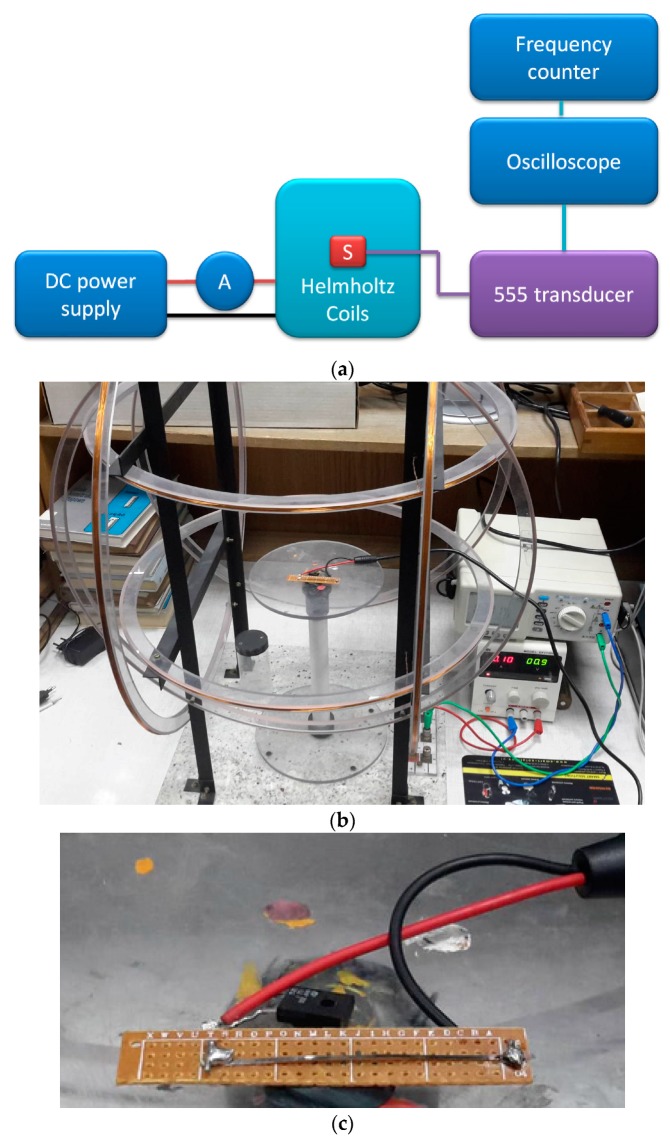
(**a**) Schematic diagram of measurement test stand for GMI magnetic field sensor calibration; (**b**) photo of actual test stand; (**c**) details of developed sensor.

The transducer was connected to experimental GMI (Giant Magneto Impedance) [29] magnetic field sensor. The schematic diagram of the measurements test stand is presented in Figure 6.

In this configuration, the 555 transducer was connected directly to ferromagnetic amorphous ribbon, 50 × 1 × 0.02 mm. The utilized material was a Co_67_Fe_3_Cr_3_B_12_Si_15_ high permeability alloy [30]. Due to the Giant Magneto Impedance effect, the ribbon impedance changes due to external magnetizing field *H.* The impedance change changes the transducer output frequency. 

Magnetizing field was set with the three-axial Helmholtz coils (ESP, Warsaw, Poland) connected to DC power supply (DF173003C, NDN, Warsaw, Poland) and ammeter (APPA 207, Warsaw, Poland). The field was proportional to the read current, and was set with 0.1 A/m uncertainty, mostly due to external interference. 

#### 2.2.4. Resistive Temperature Sensors

**Figure 7:**
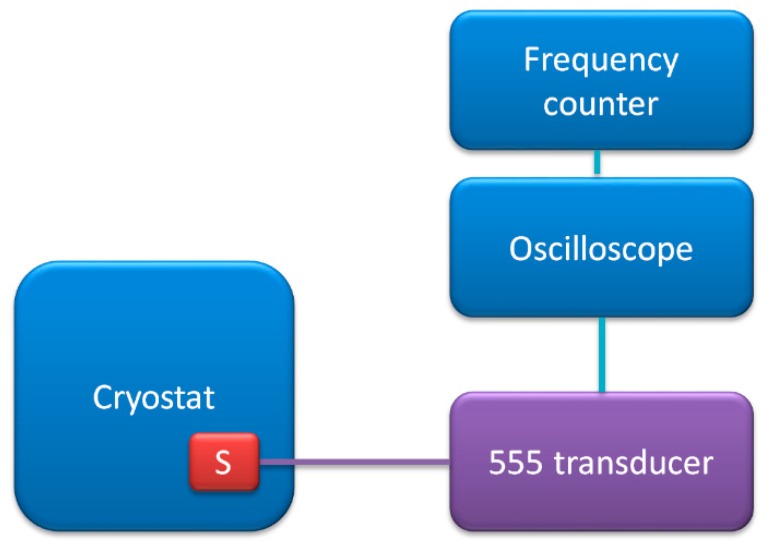
Schematic diagram of measurement test stand for resistive temperature sensor calibration.

Next, the transducer was used with thermistor resistive temperature sensor [31]. The schematic diagram of the measurements test stand is presented in Figure 7.

The NTC type thermistor (type NTC 125 K A170 2381-640-54104) was immersed in temperature-controlled silicon oil bath in cryostat (PolyScience, Niles, IL, USA). The temperature set uncertainty was 0.1 °C.

#### 2.2.5. Resistive Light Sensors

**Figure 8:**
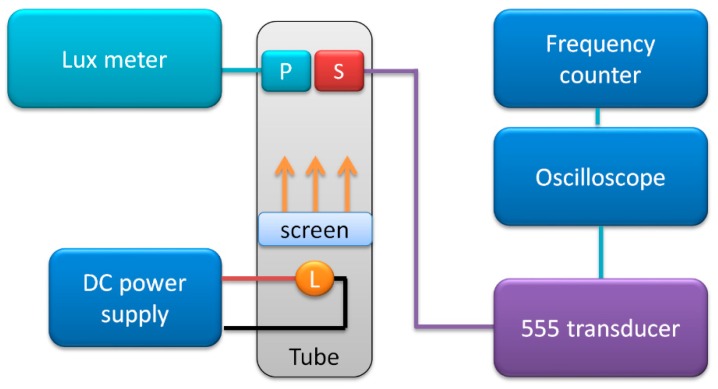
Schematic diagram of measurement test stand for resistive light sensor calibration.

Lastly, the transducer was used with photoresistor light sensor. The schematic diagram of the measurements test stand is presented in Figure 8.

The photoresistor (type 5–10 kΩ GL5616) was connected as variable impedance in the 555 transducer circuit. It was aligned with a lux meter light-sensitive probe inside an opaque pipe, with a diffusing screen in the middle and a lightbulb connected to the laboratory power supply (DF173003C, NDN, Warsaw, Poland). The current through the lightbulb was adjusted, and the lux meter (Ю-16, Mashpriborintorg, Moscow, USSR) readings were used as reference light intensity values. The set light level uncertainty was rather high, at ±10%. 

## 3. Results

The exemplary results for each type of sensor are presented below as frequency versus measured value characteristics.

### 3.1. Inductive Displacement Sensor Characteristic

**Figure 9:**
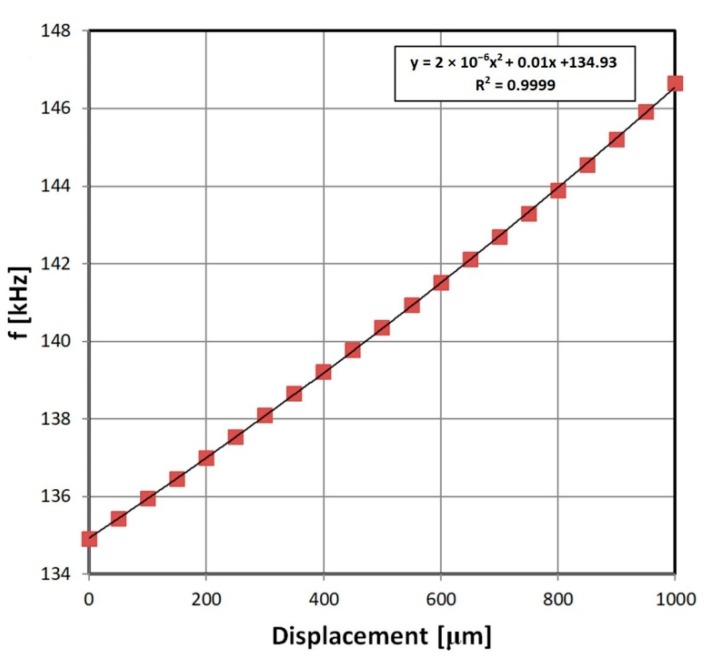
Exemplary results of inductive displacement sensor with 555 transducer output characteristics.

The inductive displacement sensor connected to 555 transducer proved to have near-linear characteristics (Figure 9). There was also no detectable hysteresis. The relative change of frequency is significant, and it shows that cheap micrometer sensors can be easily employed with presented approach. Depending on the sensor construction and frequency measurement method, 1 μm accuracy is achievable. For the presented exemplary sensor, and frequency measured with a frequency counter, the stable resolution was within 0.01% (0.1 μm) and the accuracy (mostly due to repeatability of the results) was 0.1% (1 μm). This is especially interesting given the transducer simplicity. 

### 3.2. Magnetoelastic Force Sensor Characteristic

**Figure 10:**
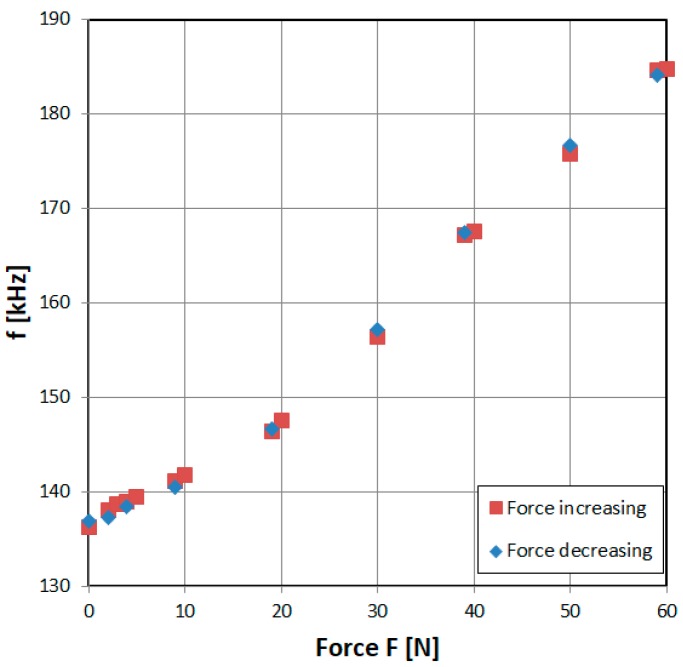
Exemplary results of magnetoelastic sensor with 555 transducer output characteristic.

The characteristics of the simplistic magnetoelastic force sensor are presented in Figure 10. The obtained stable resolution was 0.02% (0.012 N). The accuracy of the sensor however is limited to ±1% (±5% in the low force region), mainly due to the sensor’s hysteresis, which is a major and unavoidable drawback of magnetoelastic technology [32]. The repeatability was therefore limited by the hysteresis to ±1–5%. There are some advanced approaches that can be used to minimize this error [33]. On the other hand, the magnetoelastic characteristic of constructional steel is proven to be stable and temperature insensitive [34], which provides an extremely cheap (two pieces of wire + simple transducer) and robust tensile force sensor.

### 3.3. GMI Magnetic Field Sensor Characteristic

**Figure 11:**
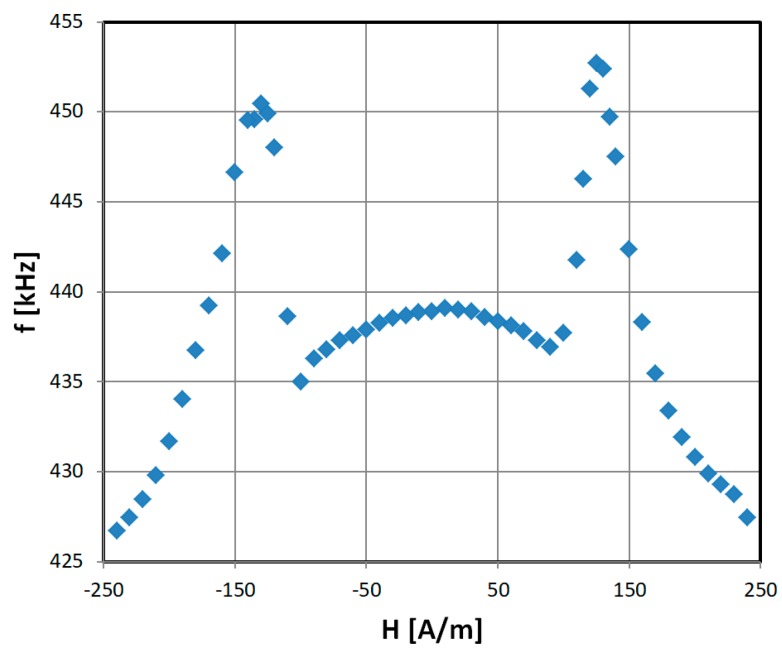
Exemplary results of GMI sensor with 555 transducer ‘batman’ output characteristic.

The transducer operation was further verified on sensors which experience impedance changes due to an external magnetic field, which is called the GMI effect. An exemplary characteristic for a Co_67_Fe_3_Cr_3_B_12_Si_15_ high permeability alloy is presented in Figure 11. Unfortunately, the characteristic is a ‘double peak’ type, which is typical for cobalt-based, annealed high permeability ribbons [17]. This complicates usage of this sensor significantly, but the same problem is common for almost all GMI technology [35]. One of the solutions is proper treatment of the sensing material [1]. Suitable measurement methods were also described when the effect was first observed [15,16]. The characteristic is slightly different from a typical GMI response due to square wave being utilized in a 555 transducer. The stable resolution in the linear regions was 0.03% (0.05 μT) and the accuracy was 0.5% (0.75 μT). The repeatability was limited mainly by external field interference to ±1%.

### 3.4. Resistive Temperature Field Sensor Characteristic

**Figure 12:**
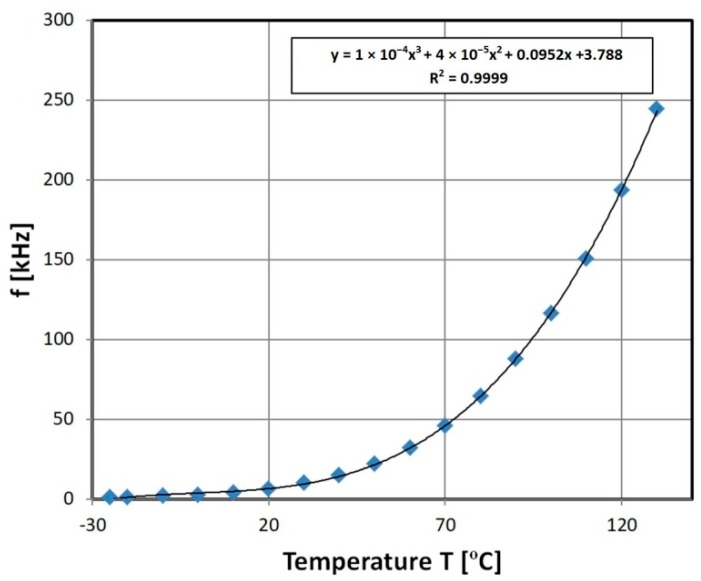
Exemplary results of N type thermistor with 555 transducer output characteristics.

The NTC type thermistor was a perfect candidate for the presented transducer, due to its exponential fall of resistivity with temperature, monotonic characteristic (in contrast to PTC type thermistors), and high initial resistance. Exemplary characteristics are presented in Figure 12. For the presented exemplary N type thermistor, the stable resolution was changing exponentially from 1% to 0.004% (0.006 °C) (in the high temperature region) and the accuracy was limited to 1–0.1% (1–0.1 °C), due to calibrating cryostat accuracy. The repeatability changes with temperature as the resolution; it was ±1% in the low temperature range and ±0.1% in higher ranges due to cryostat uncertainty.

### 3.5. Resistive Light Sensors Characteristic

**Figure 13:**
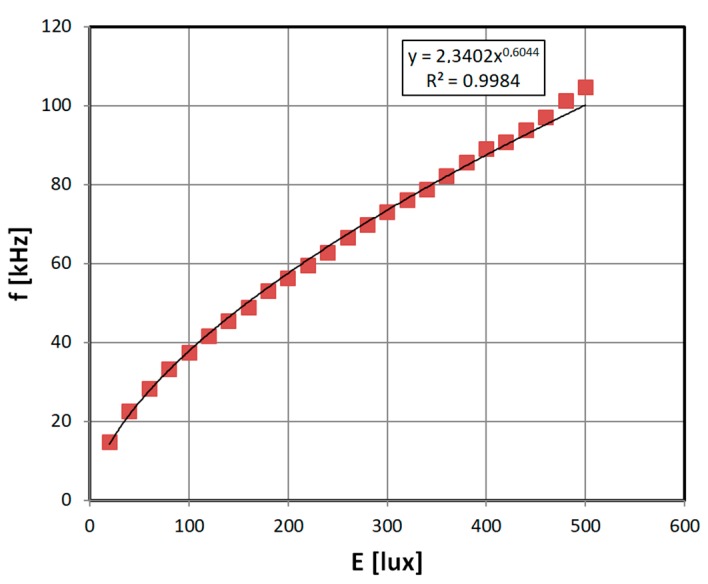
Exemplary results of photoresistor with 555 transducer output characteristic.

Lastly, the photoresistor characteristic is presented (Figure 13). The characteristic is monotonic, and allows for significant measurement resolution, outperforming typical voltage divider configuration. For the presented exemplary photoresistor, the stable resolution was 0.01% (0.05 lux), the repeatability was within 0.1%, and the accuracy was limited to 10% (50 lux), due to calibrating luxmeter accuracy.

## 4. Conclusions

The presented astable impedance-frequency converter allows for direct application of various experimental sensors in modern microcontroller-based systems. It is further supported by directly-measurable frequency output and 5 V supply/output voltages typical for systems such as Arduino systems. 

The major advantages of the transducer are universality (it accepts a wide range of input impedances, from purely resistive to inductive), exceptional simplicity, and a low cost of construction. Together with presented sensors, it can be a part of economic, automatic measurement systems for a wide range of physical quantities. 

The major drawbacks of the 555 based transducer are problems with circuit simulation (the author found no suitable SPICE software), resulting in the necessity for individual sensor type calibration. 

All in all, the presented solution is interesting as a method of fast, simple and exceptionally low-cost application of a wide range of experimental or custom-made sensors.
